# Functional feeding traits as predictors of invasive success of alien freshwater fish species using a food-fish model

**DOI:** 10.1371/journal.pone.0197636

**Published:** 2018-06-06

**Authors:** Leopold A. J. Nagelkerke, Eline van Onselen, Nils van Kessel, Rob S. E. W. Leuven

**Affiliations:** 1 Aquaculture & Fisheries Group, Wageningen University & Research, Wageningen, The Netherlands; 2 Institute for Water and Wetland Research, Department of Animal Ecology and Physiology, Radboud University, Nijmegen, The Netherlands; 3 Bureau Waardenburg B.V., Culemborg, The Netherlands; 4 Netherlands Centre of Expertise on Exotic Species (NEC-E), Nijmegen, The Netherlands; Technical University of Denmark, DENMARK

## Abstract

Invasions of Ponto-Caspian fish species into north-western European river basins accelerated since the opening of the Rhine–Main–Danube Canal in 1992. Since 2002, at least five Ponto-Caspian alien fish species have arrived in The Netherlands. Four species belong to the Gobiidae family (*Neogobius fluviatilis*, *Neogobius melanostomus*, *Ponticola kessleri*, and *Proterorhinus semilunaris*) and one to the Cyprinidae family (*Romanogobio belingi*). These species are expected to be potentially deleterious for the populations of four native benthic fish species: *Gobio gobio* (Cyprinidae), *Barbatula barbatula* (Nemacheilidae), *Cottus perifretum*, and *C*. *rhenanus* (Cottidae). Invasion success may be dependent on competitive trophic interactions with native species, which are enabled and/or constrained by feeding-related morphological traits. Twenty-two functional feeding traits were measured in nine species (in total 90 specimens). These traits were quantitatively linked to the mechanical, chemical and behavioral properties of a range of aquatic resource categories, using a previously developed food-fish model (FFM). The FFM was used to predict the trophic profile (TP) of each fish: the combined capacities to feed on each of the resource types. The most extreme TPs belonged to three alien species, indicating that they were most specialized among the studied species. Of these three, only *P*. *kessleri* overlapped with the two native *Cottus* species, indicating potential trophic competition. *N*. *fluviatilis* and *R*. *belingi* did not show any overlap, indicating that there is low trophic competition. The two remaining alien goby species (*N*. *melanostomus* and *P*. *semilunaris*) had average TPs and could be considered generalist feeders. They overlapped with each other and with *G*. *gobio* and *B*. *barbatula*, indicating potential trophic competition. This study suggests that both generalist and specialist species can be successful invaders. Since the FFM predicts potential interactions between species, it provides a tool to support horizon scanning and rapid risk assessments of alien species.

## Introduction

Invasions of aquatic alien species are increasingly recorded all over the globe and can have profound and pervasive effects on ecosystems [1–4], even to such an extent that they are considered a global threat to biodiversity and human livelihoods [5,6]. Impacts of alien species involve both direct biotic interactions, such as parasitism [7] and predation [8] as well as indirect changes through competition for resources [9,10], spread of disease and parasites [7,11], and even habitat alterations [8]. Because of the strong trophic links in the aquatic environment food-web interactions are of special interest to understand the dynamics of native and alien species [8,12,13].

One of the most extensive invasion waves is that of Ponto-Caspian species, which was facilitated by the development of the European network of water ways, shipping and ballast water [14]. Since 1992 the spread of both invertebrates and fish species has accelerated through the connection of the Danube and Rhine river basins [14,15]. In the Netherlands this has resulted in the arrival of at least four alien gobies (Gobiidae) and one gudgeon species (Cyprinidae) [16–18]. In order of first date of record: Western tubenose goby, *Proterorhinus semilunaris* (Heckel, 1837) in 2002, Whitefin gudgeon, *Romanogobio belingi* (Slastenenko, 1934) in 2004, Round goby, *Neogobius melanostomus* (Pallas, 1814) in 2004, Bighead goby, *Ponticola kessleri* (Günther, 1861), in 2007, and Monkey goby, *Neogobius fluviatilis* (Pallas, 1814) in 2009.

Invasibility is higher in low-diversity and disturbed ecosystems [12], e.g. in freshwater fish communities [19] and appears to be driven by two phenomena: the existence of “vacant” or “empty” niches and competitive interactions between native and alien species [20–22]. In the case of the Ponto-Caspian fish invasion in The Netherlands, two goby species (*P*. *kessleri* and *N*. *melanostomus*) were shown to be able to outcompete River bullhead (*Cottus perifretum*), most likely for shelter and/or food [23]. Other forms of competition have been suggested in reproduction, habitat use and resource use [24]. But either as a predator, competitor or prey item, alien species have an impact on the trophic relations within an ecosystem [12]. However, trophic competition between alien Ponto-Caspian and native fish species is not well understood, despite the existence of some diet analyses [25–28]. Moreover, the actual diet of fishes is highly dependent on resource availability in a particular context and does not necessarily reflect the competitive potential in new circumstances [29], such as in newly invaded ecosystems. Therefore the ability to predict prey use from the morphology of an alien species could be helpful in the assessment of its potential impact on the receiving ecosystem. Such an ecomorphological approach is based on the principle that the morphology of an organism is causally linked to its feeding capacities [29–31] and that morphological feeding traits that are optimal for a particular food type, may be limiting in processing other food types [29,32,33]. Several studies have explicitly investigated the relationship between morphology, ecological functioning, and invasion potential of fish [34–36]. In these studies morphology is either used as a proxy for the ecological position of a species [34], or the relationship between morphology and ecological functioning are related through a correlative approach [35].

In contrast to the aforementioned correlative approaches Sibbing and Nagelkerke [29] developed a “Food-Fish Model” (FFM) in which the functional feeding traits of fish are quantitatively linked to the capacity to feed on a suite of aquatic food types, by means of functional morphology. The FFM aims to predict which resources are likely to make a difference in case of competitive circumstances. This means that not only the differences in trophic morphology between species are shown, but also the implications of these differences for the capacity to utilize particular food resources. For that purpose resources are divided into categories that reflect the challenges they pose to their consumer (e.g. fish prey are fast, large, struggling, and easily digestible, as opposed to sessile algae, which are non-moving, small and hard to digest). The quality of the FFM depends therefore on how well resources and consumer traits can be matched [29,37], which in turn depends strongly on the knowledge of functional morphology.

The model was originally developed using a group of African carp-like fishes (Cyprinidae) and was successfully used to predict their diets [29]. In the present study the FFM was used to explore the feeding capacities of the five aforementioned alien Ponto-Caspian species *N*. *fluviatilis*, *N*. *melanostomus*, *P*. *semilunaris*, *P*. *kessleri*, and *R*. *belingi*, and four native species: Gudgeon, *Gobio gobio* (L., 1758) (Cyprinidae), Stone loach, *Barbatula barbatula* (L., 1758) (Nemacheilidae), and two bullhead species, River bullhead, *Cottus perifretum* Freyhof, Kottelat & Nolte, 2005, and Brook bullhead, *C*. *rhenanus* Freyhof, Kottelat & Nolte, 2005 (Cottidae). These native species were selected because of their assumed (partly) overlapping ecological niches and shared habitats with the alien species, and /or reported adverse effects of alien species on their populations [23,24,38]. It is discussed whether the alien species, based on their feeding capacities, could pose a threat to the native species and to what extent competitive interactions for food could play a role in this.

## Materials and methods

### Sampling

In total 85 fish (6–14 specimens per species), for which a complete morphological dataset could be collected were used (Table 1). Dead frozen specimens were provided from fish monitoring conducted in compliance to the Water Framework Directive (WFD; 2000/60/EC). Fish were caught in the period 2011–2013 in different water bodies in the Netherlands (Hollands Diep, Waal, IJssel, Zandmaas, Grensmaas, Geul, Gulp: S1 Fig), using electrofishing and seine netting, according to van Kessel et al. [10].

**Table 1:** Origin and length of fish used for morphological trait analysis.

Species	Status	Number of specimens	Standard length range (mm)	Mean (mm)	SD (mm)
*Barbatula barbatula*	Native	10	41.1–75.2	64.3	12.2
*Cottus perifretum*	Native	8	40.5–69.6	54.4	8.9
*Cottus rhenanus*	Native	9	43.8–64.6	55.2	5.5
*Gobio gobio*	Native	6	92.7–115.7	106.4	10.4
*Neogobius fluviatilis*	Alien	10	51.4–118.3	72.4	19.6
*Neogobius melanostomus*	Alien	13	46.5–99.7	69	21.6
*Ponticola kessleri*	Alien	9	68.2–95.0	80	8
*Proterorhinus semilunaris*	Alien	10	40.0–68.3	54.5	8.3
*Romanogobio belingi*	Alien	10	63.0–105.6	85.5	15.8

### Measurements

Twenty-two functional feeding traits were measured using electronic calipers (Sylvac, Switzerland) and a dissection microscope. For measurements smaller than 2 mm an eyepiece micrometer was used. All measurements followed Sibbing and Nagelkerke [29], but as the original method was developed for cyprinid fishes, some traits could not be measured for all species in this study and were left out. Some trait measurements were adapted, such as presence and absence of barbels, and the presence of oral and pharyngeal teeth (Table 2). Metric measurements were scaled by dividing them by the standard length (SL). Surface areas were divided by SL^2^, while ratios, degrees and presence / absence measurements (0/1) were not scaled. All raw measurement data on functional feeding traits are included in the supplementary material (S1 Table).

**Table 2:** Morphological traits used in the trophic profile analysis of fish species.

Morphological trait	Abbreviation / description	Unit	Scaled by
Barbels (adapted from Sibbing and Nagelkerke [29]: only absence / presence is recorded) *	Ba	presence / absence	N.A.
Body depth	BD	mm	SL
Caudal peduncle depth	CPD	mm	SL
Eye diameter	ED	mm	SL
Gape size	OGAr	mm^2^	SL^2^
Gill arch resistance	GiRL/GiRD: ratio between gill raker length and gill raker distance	ratio	N.A.
Gill raker distance	GiRD	mm	SL
Gill raker length	GiRL	mm	SL
Gut length	GuL	mm	SL
Head length	HL	mm	SL
Hyoid length	HyL	mm	SL
Lower jaw closing force efficiency	Ljin/Ljout: the ratio between input and output closing lever of the lower jaw	ratio	N.A.
Lower jaw length	LJL	mm	SL
Operculum area	OpAr: Postorbital length × Operculum depth	mm^2^	SL^2^
Oral gape axis	OGAx	degrees	N.A.
Oral teeth presence **	TOT	presence / absence	N.A.
Pharyngeal molariform teeth **	TPT2	presence / absence	N.A.
Postlingual organ width	PLOW	mm	SL
Protrusion length	ProtL: extension of the upper jaw when opening the mouth	mm	SL
Relative gape area	OGAr/Bar: ratio between oral gape area and body area (body width × body depth)	ratio	N.A.
Velocity suction capacity	HyL/LJSL: ratio between hyoid and lower jaw-suspensorium length	ratio	N.A.
Volume capacity operculum	POrL/OpD: ratio between postorbital length and operculum depth	ratio	N.A.

N.A.: not applicable; SL: standard length (SL^2^ in case of surface area). All measures follow Sibbing and Nagelkerke [29], unless otherwise stated.

* This is an adapted trait: in Sibbing and Nagelkerke [29] barbel length is measured, but because most species in this study do not have barbels, the absence or presence of barbels is used instead.

** These are new traits of tooth presence. Shape description of molariform pharyngeal teeth follows Fryer and Iles [39].

Interpretation of the functionality of the presence of teeth is based on Sibbing [40] and Sibbing and Nagelkerke [29].

### Data analysis

For each fish its morphological traits were, through functional morphology, systematically related to their effects on the capacity to utilize particular aquatic food types using the FFM. This was done in two steps as described in Sibbing and Nagelkerke [29]. First the effects of morphological traits on the capacity to eat a suite of aquatic food types were established. These effects were quantitatively expressed as positive or negative values (ranging between -2 and +2, where values of ±2 indicate stronger effects than values of ±1), or zero values when there is no functional morphological evidence for an effect. The combined values of all such effects for an aquatic resource form a hypothetical “food specialist profile” (FSP), expressing the ideal relative sizes of morphological traits to exploit that resource (S2 Table). The FSP values for each aquatic food resource were correlated with the morphological measurements of each individual fish, using Kendall’s tau correlation. The resulting series of correlation coefficients was called a trophic profile (TP), expressing the relative capacity of each individual fish to utilize each of the food resources. The values of measured morphological traits and values of FSPs, were separately standardized before correlation (subtracting the mean value of each variable and dividing by the standard deviation), resulting in a mean value of zero and a standard deviation of one for each variable, thus giving equal weight to all variables.

Principal components analysis (PCA) was performed on the standardized functional feeding traits to compare overall trophic morphology, and also on the TPs, to compare the overall capacities to utilize aquatic food resources. Mean TPs per species were calculated and clustered by species to explore differences in feeding capacities between species, and by food type to explore which food types are likely to differentiate most between the studied species.

All statistics were performed in R [41]. Clustering was performed with the pvclust package, using 10,000 bootstrap replicates and the ward.D2 option [42].

## Results

**Fig 1:**
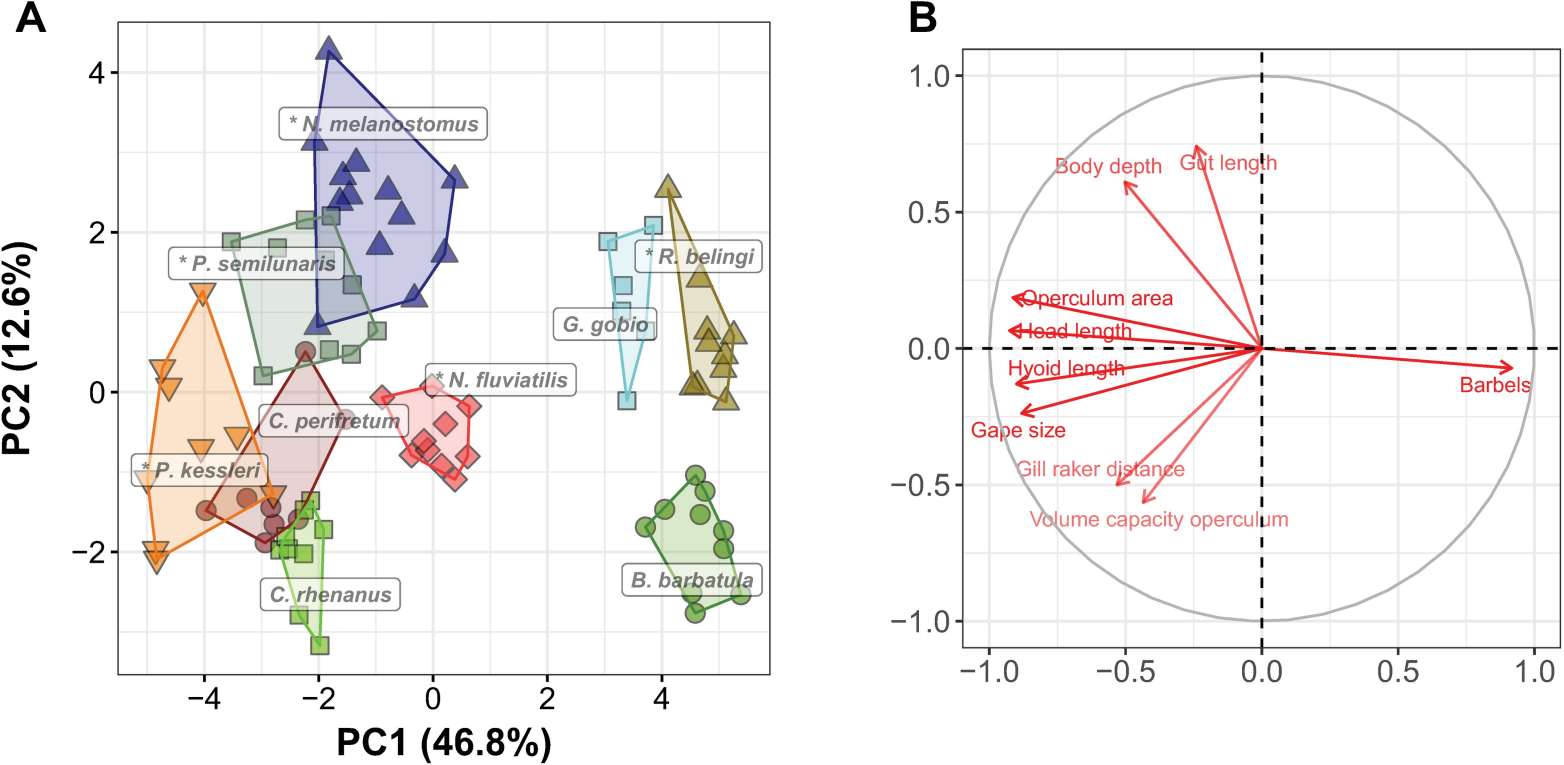
Ordination of overall trophic morphology. Principal component analysis of 22 functional feeding traits of five alien and four native fish species. In the left panel each marker represents an individual, and different symbols and colors indicate different species. Alien species are indicated with an asterisk. In the right panel the directions and sizes of the loadings of the feeding traits on the ordination are indicated (note that for clarity not all traits are shown).

The first two dimensions of the PCA ordination of the functional feeding traits represented 59.4% of the total variation and revealed that trophic morphology is generally different between species (Fig 1). There is little overlap between species, despite the presence of intraspecific variation and there appears to be no obvious sorting between native and alien species. *N*. *fluviatilis* has the most average trophic morphology, indicated by its position in the center of the ordination. The group in the right half of the ordination, consisting of native *B*. *barbatula* and *G*. *gobio*, and of alien *R*. *belingi*, is separate from all other species in the ordination, which is mostly caused by the presence of barbels and the absence of oral teeth in these three species, as well as by on average smaller gapes and operculum areas. Within this group *B*. *barbatula* is characterized by a shorter gut and a less deep body than the two gudgeon species. The group of fishes in the lower left quadrant of the ordination consists of the two native *Cottus* species and the alien *P*. *kessleri*. These species are all three characterized by large gapes and head lengths, as well as by large gill raker distances. *P*. *kessleri* is the only alien species in this analysis that overlaps with a native species. Finally, *P*. *semilunaris* and *N*. *melanostomus* are found in the top left quadrant of the ordination. They show some overlap in trophic morphology with each other, but not with any of the native species. A relatively large body depth and gut length characterizes them.

**Fig 2:**
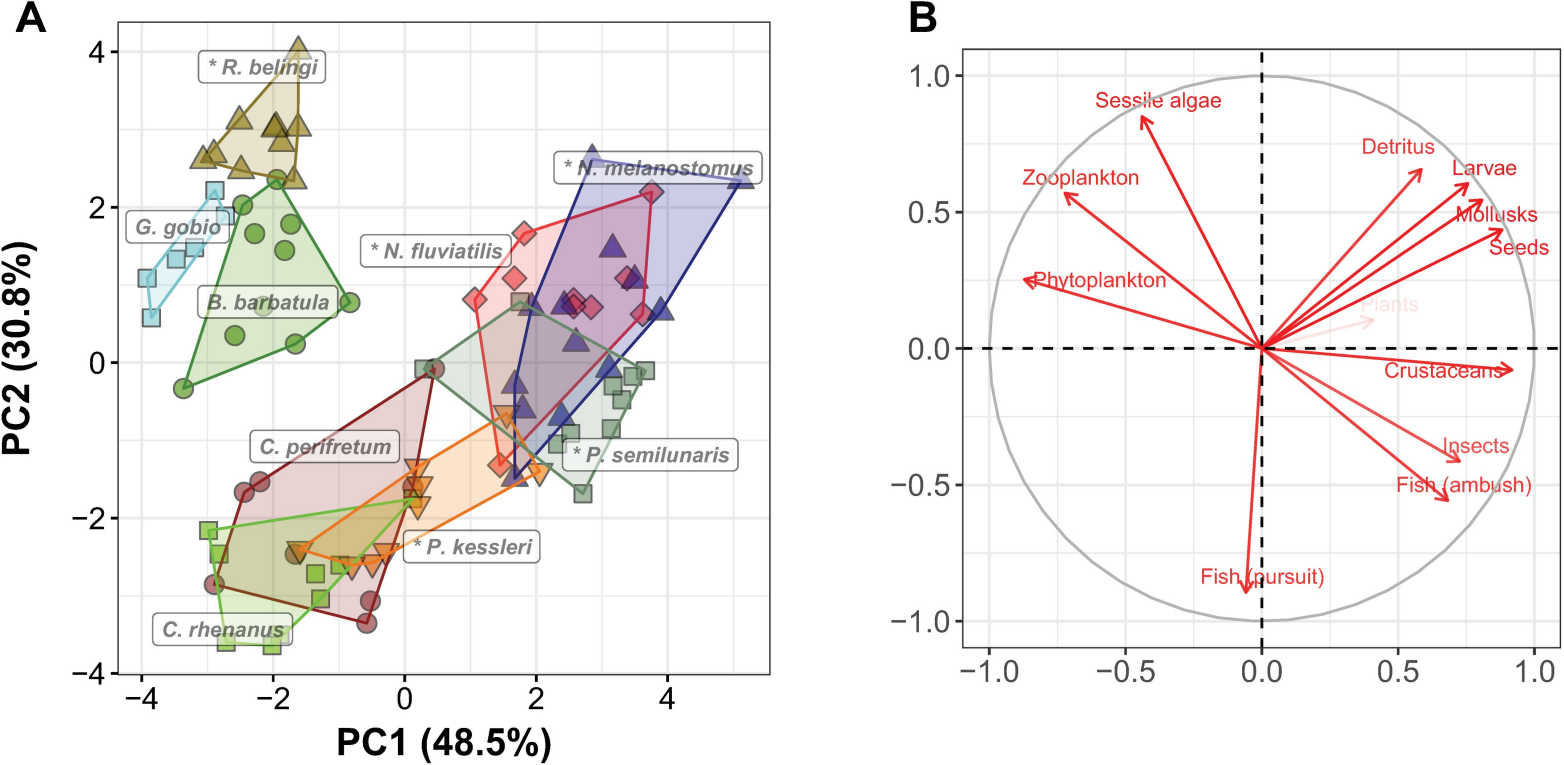
Ordination of trophic profiles. Principal component analysis of the trophic profiles of five alien and four native fish species. In the left panel each marker represents an individual, and different symbols and colors indicate different species. Alien species are indicated with an asterisk. In the right panel the directions and sizes of the loading of the food specialist profiles are indicated.

The PCA ordination of the TPs (the first two dimensions representing 79.3% of the total variation) shows a slightly different picture. TPs of the species clearly cluster, but with more intra-specific variation than the trophic morphologies (Fig 2). There are roughly three groups of TPs. The first consists of the alien species *N*. *fluviatilis*, *N*. *melanostomus*, and *P*. *semilunaris*, which are overall closest to the center of the ordination, indicating the most generalized TPs. These species show a relatively large capacity for feeding on benthic resources such as benthic insect larvae, mollusks, detritus, and crustaceans. They also strongly overlap with each other. The second group consists of native *B*. *barbatula* and *G*. *gobio* and the alien *R*. *belingi*, characterized by a higher capacity to feed on sessile algae, phytoplankton, and zooplankton, and a smaller capacity for utilizing fish and insects. Finally, there is a group consisting of the two native *Cottus* species and the alien *P*. *kessleri*, which are distinct from the other species, but overlapping with each other. These are mainly characterized by their greater capacity to feed on fish (both by ambush and pursuit hunting) and their smaller capacity for utilizing benthic resources, plankton, and sessile algae.

**Fig 3:**
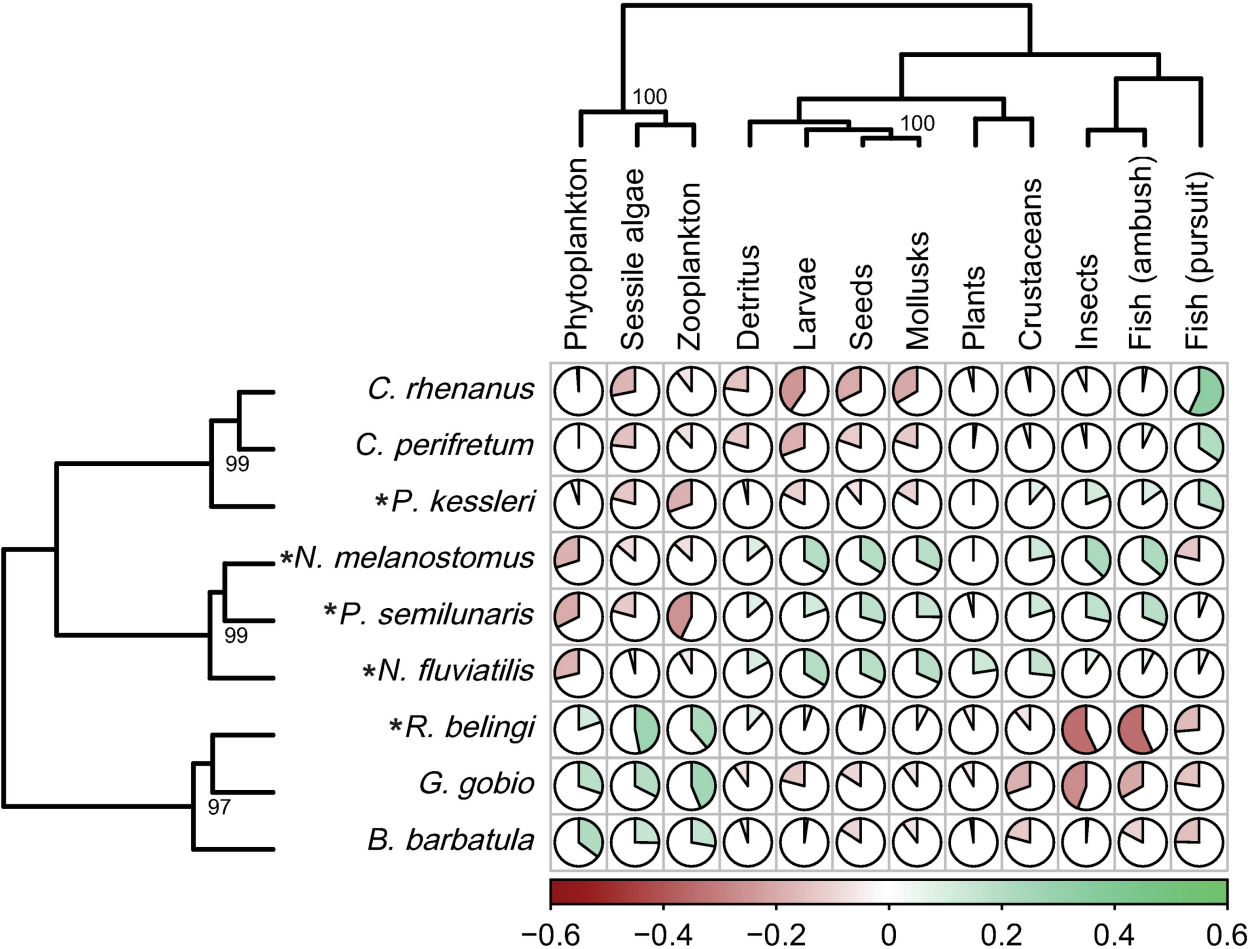
Mean trophic profiles per species. Capacities of species to utilize aquatic food types, expressed as the mean trophic profiles per species. Pies indicate the mean correlations of a species’ morphology with the hypothetical profiles for each food resource specialist (red indicate negative, green positive correlations). Trophic profiles were both clustered for species and food types. Numbers in the tree diagrams are significant (>95%) bootstrap values. Alien species are indicated with an asterisk.

It is striking that the extreme borders of both the trophic morphology and the TP spaces are mostly occupied by alien species, but that they also occur in the center. This is confirmed by the clustering of the mean TP per species (Fig 3), which shows the same three species groups, associated with three food resource groups.

## Discussion

Invasion success is hypothesized to be related to two phenomena: the existence of “vacant” or “empty” niches and superior competitive abilities of the alien invader [20–22]. We found that either of these phenomena are plausible in case of the invasion of alien Ponto-Caspian fish species in Europe. When comparing the functional trophic morphology of five alien and four native species we found that they were all distinct, with only little overlap (Fig 1), suggesting that the feeding capacity of these species is different, providing sufficient opportunity for resource partitioning and for successful invasions [34]. When interpreting these differences more directly towards differences in the capacity to utilize particular aquatic resources, more overlap between the species was seen, not only between alien and native species, but also between different aliens and different natives (Fig 2). Such overlap suggests similar trophic capacities of individuals and species, but also potential competitive interactions. Most extreme TPs are of alien species, suggesting that they are more specialized for particular food types (e.g., *R*. *belingi* for sessile algae, *P*. *kessleri* for fish, and the other three alien gobies for crustaceans). However, some specimens of especially *P*. *semilunaris* and *N*. *fluviatilis* have an average TP, suggesting that they are generalist feeders. Also in earlier studies both diet generalists and specialists were found to be successful invaders [43,44]. Similarly, two species from this study, *P*. *kessleri*, which is predicted to be a more specialist feeder in comparison to the more generalist *N*. *melanostomus*, and of which independent diet analyses supports this prediction [27], are both successful invaders and shown to be associated with the decrease of native *C*. *perifretum* [23].

The results of this study may be influenced by methodological choices that were made. Brandner et al. [45] found that different sampling methods can result in size or sex bias in the caught specimens. However, in our case the standardized combination of electrofishing and seine netting, which is performed in compliance with requirements for ecological status assessments according to the European Water Framework Directive (WFD), most probably yielded fish samples that can be considered representative for the populations. Therefore, our results are unlikely to be biased by the sampling methodology.

More importantly, the number of functional traits, but also the number of aquatic food resources that are distinguished can affect the accuracy of predicting food use and potential resource partitioning from morphology [46]. However, gradually a consensus on relevant functional traits of fish feeding emerges [29,46–48]. Moreover, as all aspects of the feeding sequence–from detection to digestion–are represented in the selected functional traits [40], the ordination of species and specimens within functional trait space (Fig 1) can be considered reliably reflecting trophic similarities. The crucial step in the FFM in comparison to other studies of the relationships between morphology and trophic performance in invasion success, is the explicit incorporation of direct causal links between functional traits and the capacity to feed on particular resources, rather than looking for a *post hoc* correlation between resource use and morphology. The aim is to predict not only the extent of similarity among species, but also to indicate which resources are likely to make a difference in case of competitive circumstances [29]. For that purpose resources are divided into categories that reflect the challenges they pose to the consumer (e.g. fish prey are fast, large, struggling, easily-digestible prey, as opposed to sessile algae, which are non-moving, small and hard to digest). The quality of the FFM depends therefore on how well resources and consumer traits can be matched [29,37], which in turn depends strongly on the knowledge of functional morphology. Refining the resource categories will most likely not make the model more reliable. In an extensive study on the relationships between morphology and trophic guilds of Mediterranean fishes Albouy et al. [46] found that no more than seven trophic guilds could be reliably distinguished. Different resource categories can partly overlap in the challenges they pose to consumers and the optimum trophic morphologies for dealing with such resource categories can therefore also be partly similar. Thus, the greater overlap between species in the ordination of the TPs (Fig 2) most likely reflects a real biological phenomenon such as the similarity in traits of different resources and not only a consequence of methodological imperfection. In this study this overlap results in roughly three resource groups (fish and insects, phytoplankton and zooplankton, all other resources) and three consumer groups, that are roughly, but not completely linked (Fig 3).

To what extent can the FFM approach be helpful in the assessment of invasion risk of alien species? As it indicates the capacity for resource utilization in relation to other species, it is potentially more useful than methods based on comparing actual diets. This is especially the case for species such as Ponto-Caspian gobies, which have broad and adaptable diets, depending on environmental conditions and food availability in (newly) invaded areas [26–28,49]. Even when an alien species is predicted to be a superior competitor for a particular resource, it does not mean that trophic competition will actually take place. If food is abundant it may not occur and even when there is a preference for the same food type between species, food partitioning without competition is possible, for instance because of habitat partitioning [50,51]. Moreover, morphological constraints can, to some extent, be compensated for by behavioral adaptations which could increase niche breadth [29,52], thereby decreasing or increasing competitive interactions. Behavioral aspects could also be underlying the phenomenon of facilitative interactions [53], which means that it is easier for new species to become invasive when others have already successfully established. For instance, in the Netherlands several Ponto-Caspian amphipod species already established before the first gobies were recorded [54,55]. It is possible that Ponto-Caspian gobies have a competitive advantage over native species in utilizing this resource because of their common evolutionary history in their native range [4].

In addition to species differences, this study also shows that there is intra-specific variation in the trophic morphology and TPs, with some extreme individuals in all species. Such individuals could have a selective advantage over others, both of other and of their own species. The individual trait utility hypothesis of Cerwenka et al. [56] even suggests that individuals carrying extreme trait values could be driving successful invasions. In *N*. *melanostomus* it was found that individuals at the invasion front were different from those in longer established areas, mostly in traits related to size, growth, and feeding (the “bigger is better” hypothesis), increasing their competitive capability [57,58]. Therefore incorporating intra-specific variation in ecomorphological analyses to assess invasion risk is highly recommended.

Of course trophic interactions are not the only mechanism that could favor alien over native species. Life history traits, such as nest-guarding, high reproductive output [59,60], and alternative reproductive tactics [61], the use of shelters [10,23,59], swimming performance [36], and aggressive behavior [24,60] can all be of importance and are not included in this analysis. However, trophic interactions are always of importance and despite its limitations, the use of the FFM could provide a relatively simple and fast tool to support rapid assessments of invasion risk.

## Conclusions

This study shows that the trophic morphologies of five Ponto-Caspian fish species that recently invaded The Netherlands are distinct from each other and also from almost all studied native species. The feeding capacities of some aliens, derived from morphology, overlapped with native species, indicating potential competitive trophic interactions. Other alien species have more extreme feeding capacities, which potentially indicate their capacity to fill “vacant niches”. This study suggests that both generalist (e.g. *N*. *melanostomus*) and specialist species (e.g. *P*. *kessleri*) can be successful invaders.

## Supporting information

S1 Fig Sampling locations. 1: Hollands Diep; 2: Waal; 3:IJssel; 4: Zandmaas; 5: Grensmaas; 6: Geul; 7: Gulp.
(PDF)

S1 Table Raw measurement data on functional feeding traits. (XLSX)

S2 Table Food specialist profiles. Effects of the sizes of morphological traits on the capacity to feed on different aquatic food types. Effects can be negative, positive or neutral (0). For velocity suction capacity, relative gape area, and relative body depth an optimum instead of a continuous relationship was used, a deviation from the optimum was seen as negative (or neutral) for these traits. The method follows Sibbing and Nagelkerke [29].
(PDF)
