# Supplementary figures and images for: Functional feeding traits as predictors of invasive success of alien freshwater fish species using a food-fish model
Source: PLoS One. 2018 Jun 6;13(6):e0197636. doi: 10.1371/journal.pone.0197636 (PMC5991376; doi:10.1371/journal.pone.0197636)

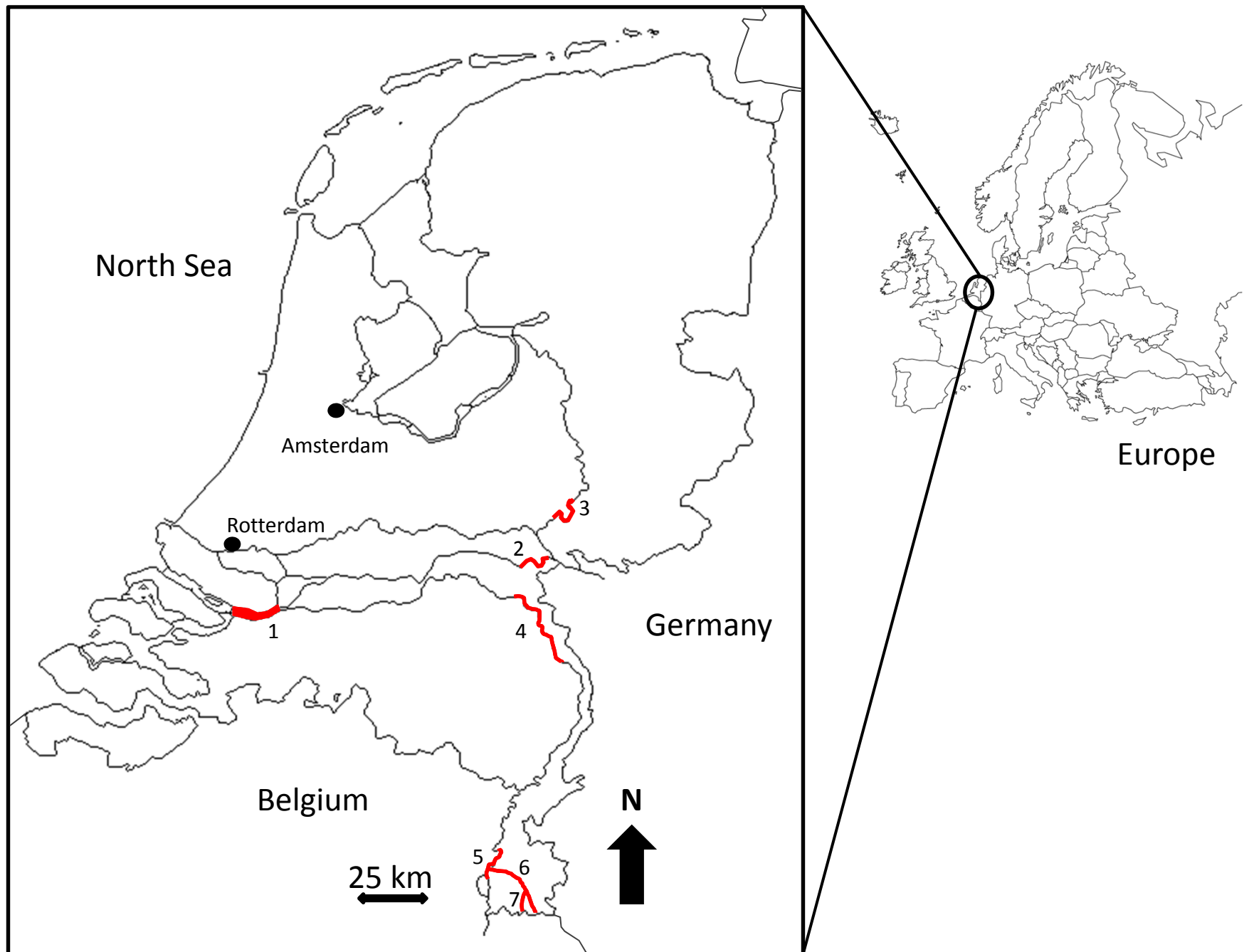

Supplement: S1 Fig — 1: Hollands Diep; 2: Waal; 3:IJssel; 4: Zandmaas; 5: Grensmaas; 6: Geul; 7: Gulp. (PDF) [file pone.0197636.s001.pdf]
