# Supplementary material for: Functional feeding traits as predictors of invasive success of alien freshwater fish species using a food-fish model
Source: PLoS One. 2018 Jun 6;13(6):e0197636. doi: 10.1371/journal.pone.0197636 (PMC5991376; doi:10.1371/journal.pone.0197636)
Supplement: S2 Table — Effects of the sizes of morphological traits on the capacity to feed on different aquatic food types. Effects can be negative, positive or neutral (0). For velocity suction capacity, relative gape area, and relative body depth an optimum instead of a continuous relationship was used, a deviation from the optimum was seen as negative (or neutral) for these traits. The method follows Sibbing and Nagelkerke [29]. (PDF) [file pone.0197636.s003.pdf]

S2 Table. Food specialist profiles

Effects of the sizes of morphological traits on the capacity to feed on different aquatic resources. Effects can be negative, positive or neutral (0). For velocity suction capacity an optimum instead of a continuous relationship was used, a deviation from the optimum was seen as negative (or neutral) for these traits. The combined values of all effects for an aquatic resource form a so-called "food specialist profile". The method follows Sibbing and Nagelkerke (2001).

Resource – feeding mode / Morphological trait	Phytoplankton – townet	Phytoplankton - pump	Algae - scraping	Plants - biting	Seeds - particulate	Detritus - particulate	Microcrustaceans - townet	Microcrustaceans - pump	Crustaceans - diverse	Benthic insect larvae / worms - particulate	Insects - diverse free-living	Mollusks - particulate	Fish - pursuit	Fish - ambush
Barbels	0	0	0	0	0	2	0	0	0	2	0	0	0	0
Body depth	-1	0	0	0	1	1	-1	0	0	1	0	1	-2	0
Caudal peduncle depth	-1	0	0	0	0	0	-1	0	0	0	1	0	-2	2
Eye diameter	0	0	0	0	0	0	0	1	0	0	0	0	0	0
Gape size	2	1	0	0	0	-1	2	1	1	-1	0.5	0	2	1
Gill arch resistance	2	2	0	0	0	0	2	2	0	0	-0.5	0	-2	-1
Gill raker distance	-2	-2	-1	0	0	-1	0	-2	0	0	0.5	0	1	1
Gill raker length	2	2	1	0	0	1	2	2	0	0	-1	0	-2	-2
Gut length	1	1	1	0.5	0	2	0	0	0	0	0	0	0	0
Head length	1	1	-1	-1	0	0	1	1	0	0	1	0	1	2
Hyoid length	0	0	0	0	0	0	0	0	0	0	0	0	0	1
Lower jaw closing force efficiency	0	0	1	2	0	0	0	0	0	0	-1	0.5	-2	-2
Lower jaw length	0	0	-1	-2	0	0	0	0	0	0	1	0	2	2
Operculum area	0	1	0	0	0	0	0	1	0	0	1	0	0	2
Oral gape axis	-1	-1	0	0	0	1	-1	-1	0	1	0	0	-1	0
Oral teeth presence	0	0	0	1	1	1	0	0	1	1	1	1	1	1
Pharyngeal molariform teeth	1	1	1	1	1	0	1	1	1	0	0	1	0	0
Pharyngeal papilliform teeth	1	1	1	1	1	0	1	1	1	0	0	1	0	0
Postlingual organ width	0	0	0	1	0.5	0	0	0	1	0	1	0.5	2	2
Protrusion length	0	0	0	-2	1	2	0	0	1	2	1	1	0	2
Relative gape area	1	0	0	0	0	0	1	0	0	0	0	0	1	0
Velocity suction	0	0	0	0	0	0	0	0	0	0	0	0	0	-1
Volume capacity operculum	0	1	0	0	0	0	0	1	0	0	1	0	1	2

Reference

Sibbing, F. A. & Nagelkerke, L. A. J. (2001). Resource partitioning by Lake Tana barbs predicted from fish morphometrics and prey characteristics. *Reviews in Fish Biology and Fisheries* **10**, 393-437.
